# Fatal injuries while under the influence of psychoactive drugs: a cross-sectional exploratory study in England

**DOI:** 10.1186/1471-2458-6-148

**Published:** 2006-06-06

**Authors:** Adenekan Oyefeso, Fabrizio Schifano, Hamid Ghodse, Kathryn Cobain, Ruth Dryden, John Corkery

**Affiliations:** 1 National Programme on Substance Abuse Deaths, Division of Mental Health, Medical School, St George's, University of London, London, SW17 0RE, UK

## Abstract

**Background:**

Studies of drug-related mortality rarely describe fatal injuries due to psychoactive drug intoxication (FIUI). The main aim of this study was to determine the nature, extent and pattern of FIUI.

**Methods:**

This observational study covered the period January 1999 to December 2001. Data were provided by members of a study panel of coroners in England using a standard protocol. Sources of data for this study included autopsy protocols, death certificates, hospital records, police reports, toxicology reports and inquest transcripts. Inclusion criteria for this were (i) the mention of one or more psychoactive substances as contributing to fatality; and (ii) the presence of a Controlled Drug at post mortem.

**Results:**

A total of 3,803 drug-related deaths of persons aged 16–64 years were reported by the study panel during the three-year period. The study panel accounted for 86% of drug-related deaths in England in this period. There were 147 FIUI cases (119 males, 28 females), giving a proportionate mortality ratio of approximately 4%. The majority of FIUI cases (84%) were aged 16–44 years, with a median age at death of 33 years (Quartile deviation = 7). Fifty-six percent of FIUI occurred in urban areas of England.

The population of the study jurisdictions aged 16–64 years contributed 49,545,766 person-years (py) to the study, giving an annual crude rate of 3/1,000,000 person-years (py). Rates for male and females were 4.9 and 1.1/1,000,000 py respectively, giving a male/female rate ratio of 4.5 (95%CI = 2.9–6.8).

The rates of intentional and unintentional FIUI were 2 and 1/1,000,000 py respectively. The leading mechanism for intentional FIUI was suffocation while the predominant mechanisms in unintentional FIUI were road traffic accidents and falls. There is a significant difference in the pattern of drug-specific risk between FIUI and fatal poisoning. Risks of intentional FIUI are elevated among Black and Minority Ethnic groups.

**Conclusion:**

There are differences in the nature, extent and pattern of intentional and unintentional FIUI that should necessitate targeted prevention strategies. Also, there is an opportunity for cross-discipline collaboration between injury prevention specialists and substance abuse/mental health specialists.

## Background

Many studies have shown that premature mortality among illicit drug users is often due to toxicity or overdose. Ghodse, Oyefeso and Kilpatrick [1] reported that about 48% of deaths among opiate addicts were due to drug overdose, either accidental or deliberate. There seems to be less interest in examining the nature, extent and pattern of fatal injuries that have occurred while intoxicated on drugs with or without alcohol.

In a large United States study of post-mortem cases of fatal injury, Hood, Ryan, Monforte and Valentour [2] reported that illicit drugs, especially cocaine, were implicated in 27% of cases. MacDonald, Anglin-Bodrug, Mann, Erickson, Hathaway, Chipman and Rylett [3] have estimated that the proportion of drugs mentioned in injuries, in general, ranged between 0% and 55%. The average estimated proportion of drugs mentioned in fatal injuries was 5%, drivers fatal; 32%, self-inflicted-fatal; and 28% general fatal.

Very few studies in the United Kingdom (UK) [4,5] have examined the pattern of fatal injuries under the influence of psychoactive drugs (FIUI). These studies have focused mainly on road traffic accidents (RTA) that are mainly unintentional in manner. Turnbridge, Keigan and James [5] reported a significant increase, over a 10-year period, in the number of RTA fatalities where psychoactive drugs had been consumed. A similar trend was reported in a 10 year-Spanish study of RTA fatalities under the influence of alcohol and other psychoactive substances [6]. Fatal intentional injuries under the influence of psychoactive drugs are even less reported in the literature, with some of these coming from the United States (US) [7,8].

Therefore, the main aim of this study was to determine the nature, extent and pattern of fatal injuries while under the influence of psychoactive drugs (FIUI). The study objectives were as follows:

• To determine the prevalence of FIUI

• To examine age, gender and ethnicity distribution of FIUI

• To identify the pattern of psychoactive drugs implicated in FIUI

• To examine the mechanisms of FIUI and their relationship to classes of psychoactive drugs

• To examine the influence of age, gender and ethnicity on FIUI

• To compare the distributions of FIUI and psychoactive drug poisoning only deaths or non-FIUI

• To examine the difference in the pattern of intentional and unintentional FIUI

Fatal injuries under the influence of psychoactive drugs are defined as cases where the fatal injury was not directly caused by poisoning, but by other means, e.g. drowning, road traffic accidents, etc, while under the influence of a psychoactive drug. In terms of manner of death, FIUI can be classified as intentional (e.g., suicide by hanging while under the influence); unlawful killing; unintentional (e.g., road traffic accidents) and undetermined.

## Methods

This observational study covered the period January 1999 to December 2001. Seventy-seven coroner jurisdictions in England formed the study panel, which accounted for 73% of all coroners' jurisdictions in England. According to the Office for National Statistics criteria for rural-urban classification [9], more than half (53%) of the study panel were in rural areas; 23% in urban areas; while 23% covered both urban and rural areas. The criterion for inclusion in the study was the provision of routine drug-related death data consistently to the National Programme on Substance Abuse Deaths (npSAD) over the three-year period of study. Members of the study panel used a standard protocol to provide data. This protocol requires coroners in England to provide data on cases of drug-related fatalities. A case is defined as one where any of the following criteria are fulfilled at a completed inquest, fatal accident inquiry or similar investigation in England: (i) one or more psychoactive substances directly implicated in fatality; (ii) history of dependence or abuse of psychoactive drugs; or (iii) presence of a controlled drug at post-mortem [10,11]. Alcohol-related cases were included only if alcohol was implicated in fatality in combination with other psychoactive substances and/or where levels of other psychoactive drugs were detected at post-mortem.

In England, the coroner whose primary function is to determine the circumstances and cause of death investigates deaths from non-natural causes, or sudden deaths. The coroner also examines any criminal involvement. The coroner requests a post mortem to be conducted by a pathologist. In each case, the pathologist determines the drugs that contributed to fatality. Given, the variation in individual levels of tolerance, the threshold for intoxication is determined on a case-by-case basis. The coroner may not request toxicological examinations if there is sufficient evidence of the likely causes of death. However, routine toxicological analysis is requested when (i) there is strong presumptive evidence that a non-natural death is drug-related; (ii) the death is suspected poisoning suicide; (iii) a fatal injury occurs as a result of a road traffic accident; and (iv) the cause of death is unclear [12]. Due to the variations in the location and resources available to coroners, toxicological specimens are analysed in different laboratories often with similar protocols.

Additional information on the circumstances of death is collected by the coroner from a variety of sources such as the police, treatment centres, relatives, general practitioner (GP), friends and other available witnesses. The coroner then holds an inquest and arrives at a verdict after considering all available evidence in the pathologist's report, autopsy protocols, hospital records, police reports, pathology and toxicology reports, and inquest transcripts.

Inclusion criteria for this study were (i) the mention of one or more psychoactive substances as contributing to fatality; and (ii) the presence of a Controlled Drug at post mortem. In the UK, the term 'controlled drugs' refers to drugs that are listed under the Misuse of Drugs Act, the main piece of legislation covering drugs; it categorises drugs as class A, B and C, according to the degree of abuse liability and associated health and social consequences.

The study excluded non-intoxicated passengers killed in a road traffic accident where an intoxicated person was driving and other non-intoxicated victims of accidents, e.g., pedestrians hit by an intoxicated driver. All cases in the study died before any sustained treatment was provided, dying instantly, on the way to hospital; or on arrival at hospital.

The study variables included age, gender and ethnicity of decedents, person-years at risk, location and setting of death, and psychoactive substances implicated in fatality. Drug-specific fatality was reported when a drug was implicated in fatal injury either alone or in combination with other drugs. Cases aged 16–64 years were included in this study given that this age range is known to be most at risk of a drug-related fatality [1].

### Research Ethics Committee approval

The Central Office for Research Ethics Committees (COREC), the national body responsible for upholding research ethics in the UK confirmed that approval was not required for this study.

### Data analysis

We calculated FIUI rate (per 1,000,000 person years at risk) using the estimated mid-year population distribution of the coroners' jurisdictions in the study panel. Person-years were calculated using the life table method. The influence of age and gender on FIUI was examined using Poisson regression. Chi square test, mortality odds ratios (MOR) and their 95% confidence limits were used in comparisons involving categorical variables. The Mann Whitney U test was used to examine the difference between the average age at death in FIUI and non-FIUI cases. Poisson regression analysis was undertaken using PEPI [13]. All other analyses were undertaken using SPSS version 13.

## Results

A total of 3,803 drug-related deaths of persons aged 16–64 years were reported by the study panel during the three-year period. The study panel accounted for 86% of drug-related deaths in England, in persons aged 16–64 years, over the three-year period of study. There were 147 FIUI cases (119 males, 28 females), giving a proportionate mortality ratio of approximately 4%. The majority of FIUI cases (84%) were aged 16–44 years, with a median age at death of 33 years (Quartile deviation = 7). Fifty-six percent of cases occurred in urban areas of England.

### Manner of FIUI

Intentional (61%, 90) and unintentional (35%, 52) accounted for the majority of FIUI. Other categories were unlawful killing (1%) and undetermined cases (4%).

### Mechanism of FIUI

**Figure 1:**
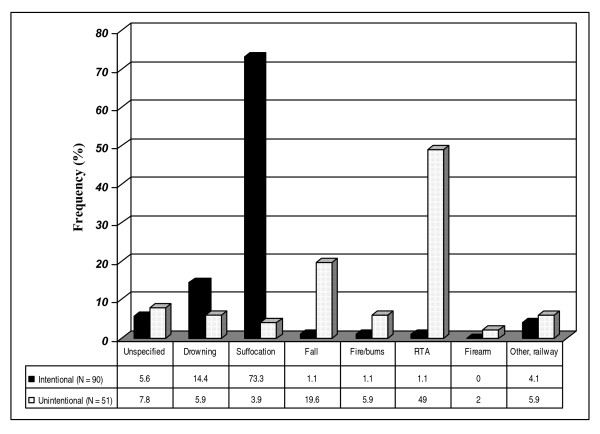
FIUI mechanisms.

The leading mechanisms of intentional FIUI were suffocation, mainly hanging (73%), and drowning (14%); while RTA (49%) and falls (20%) were the predominant mechanisms of unintentional FIUI (Figure 1).

### Implicated drugs

**Figure 2:**
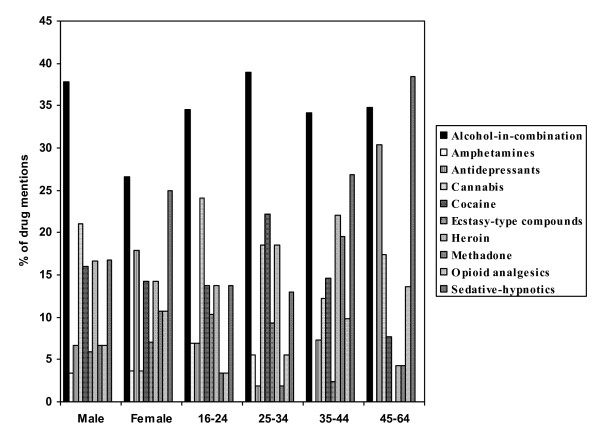
Drugs implicated in FIUI by gender and age.

The most frequently mentioned drugs in FIUI were alcohol-in-combination with other psychoactive drugs (36%); sedative-hypnotics (18%); cannabis (18%); heroin (16%) and cocaine (16%). In both males (38%) and females (27%), alcohol-in-combination was the principal drug implicated. Additionally, in males the role of the following drugs in fatality was prominent: Cannabis (21%); sedative-hypnotics (17%); heroin (17%); and cocaine (16%). The corresponding drugs in females were: Sedative-hypnotics (25%); antidepressants (18%); heroin (14%); cocaine (14%); opioid analgesics (11%) and methadone (11%) (Figure 2).

Alcohol-in-combination was the principal drug implicated across all age groups. However, there were variations in the pattern of involvement of other drugs. Besides alcohol, cannabis was the most frequently implicated drug among cases aged 16–24 years; cocaine among those aged 25–34 years; while sedative-hypnotics and antidepressants were the principal drugs implicated in the older age groups (Figure 2).

In 45% of cases, FIUI involved multiple drugs (excluding alcohol-in-combination). In intentional FIUI, 23% of fatalities involved a combination of two drugs. A combination of three drugs accounted for 7% while a combination of four drugs accounted for 4% of intentional FIUI. In unintentional FIUI, 33% of fatalities involved a combination of two drugs. Other fatalities involved a combination of three (14%) and four (14%) drugs (Table 1).

Alcohol-in-combination was implicated in 31 intentional FIUI cases. In these cases, the following drugs were detected at post-mortem: Antidepressants (1); amphetamine (2); opioid analgesics (5); heroin/morphine (3); methadone (2); ecstasy-type compounds (3); cannabis (5); cocaine (1); hypnotics (6); and anti-epileptics (3).

Alcohol-in-combination was implicated in 22 unintentional FIUI cases. Drugs detected at post-mortem included cannabis (6); cocaine (6); heroin/morphine (3); sedative-hypnotics (4); methadone (4); opioid analgesics (1); ecstasy-type drugs (2); antipsychotics (1); and hallucinogens (1).

### Implicated drugs in intentional and unintentional of FIUI

**Figure 3:**
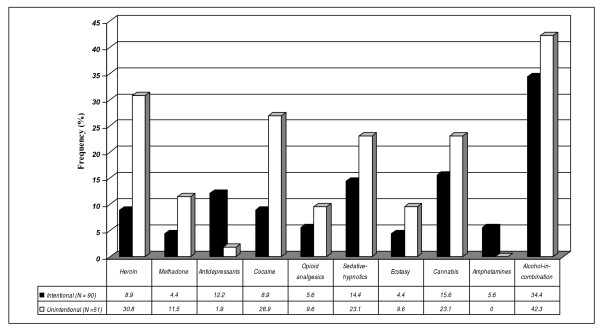
Implicated drugs in FIUI.

The pattern of drug-specific fatality was somewhat different between intentional and unintentional FIUI. Antidepressants were more frequently implicated in intentional FIUI while alcohol-in-combination, heroin/morphine, methadone, cocaine, sedative-hypnotics, ecstasy and cannabis were more frequently implicated in unintentional FIUI (Figure 3).

### Implicated drugs and FIUI mechanisms

**Figure 4:**
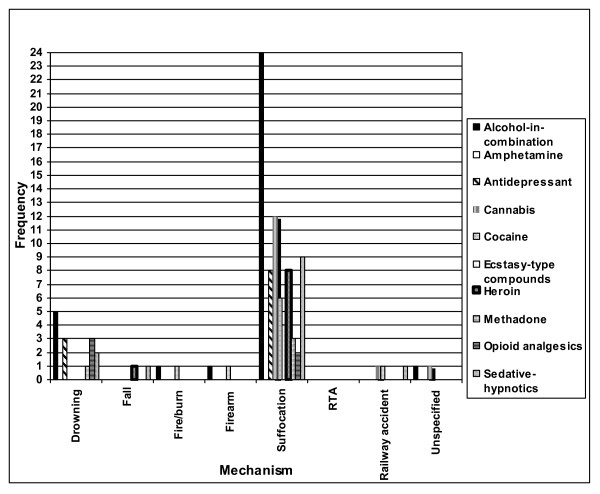
Intentional FIUI mechanisms and implicated drugs.

Involvement of psychoactive drugs in FIUI followed a polydrug use pattern. However, there were variations in this pattern. In intentional FIUI cases, all main psychoactive drug classes featured in suffocation, with alcohol-in-combination and cannabis intoxication featuring more frequently than others. Other mechanisms were more drug-specific. Drowning was mainly associated with toxicity due to alcohol-in-combination; antidepressants and opioid analgesics (Figure 4).

In unintentional FIUI cases, alcohol-in-combination and cannabis featured prominently in RTAs; alcohol-in-combination was the predominant drug in falls. Heroin/morphine featured prominently in drowning and RTAs; while sedative-hypnotics were associated more with RTAs than other mechanisms.

### Differences between FIUI and non-FIUI cases

Intentional FIUI cases were compared to intentional poisoning only cases while unintentional FIUI cases were compared to accidental poisoning only cases. Intentional FIUI cases were more likely to have the following drugs implicated in fatality: Alcohol-in-combination (MOR = 1.5, 95% CI = 1.1–2.1); amphetamines (MOR = 4.9, 95% CI = 1.6–15.2); cocaine (MOR = 11.0, 95% CI = 3.7–32.9); and ecstasy-type drugs (MOR = 5.5, 95% CI = 1.5–20.1). Intentional FIUI cases were less likely to have opioid analgesics implicated in fatality (MOR = 0.2, 95% CI = 0.06–0.4).

Unintentional FIUI cases were more likely to have involved alcohol-in-combination (MOR = 1.1, 95% CI = 1.1–2.2); cannabis (MOR = 18.6, 95% CI = 10.2–38.7); cocaine (MOR = 4.3, 95% CI = 2.6–7.0); ecstasy-type drugs (MOR = 4.3, 95% CI = 1.8–10.3) and sedative-hypnotics (MOR = 2.0, 95% CI = 1.2–3.3). Altogether, alcohol-in-combination, cocaine and ecstasy-type drugs were more likely to be implicated in FIUI than in fatal poisoning only (Table 2).

#### Gender

Approximately 19% (N = 90) of intentional FIUI cases were females compared to 40% (N = 606) of intentional poisoning cases. The corresponding figures for male intentional FIUI cases was 81% and 60% male intentional poisoning cases (χ^2 ^= 14.5, df = 1, p = 0.0001). This finding suggests that among females, intentional poisoning was a more likely method of drug-related fatality than intentional FIUI. Gender distributions of unintentional FIUI (Male 83%; Female = 17%) and unintentional poisoning (Male = 80%; Female = 20%) were similar ((χ^2 ^= 0.28, df = 1, p = 0.60)

#### Age

There was a significant difference in the average age at death of intentional FIUI cases (median age = 33 years) and that of intentional poisoning cases (median age at death = 40 years) (Mann Whiney U = 19312.0, p = 0.0001). There was no significant difference between the median age at death of unintentional FIUI cases (32 years) and unintentional poisoning (33 years).

### The influence of gender and age on FIUI

The population of the study jurisdictions aged 16–64 years contributed 49,545,766 person-years (py) to the study. This resulted in a FIUI rate of 3/1,000,000 py.). Rates for male and females were 4.9 and 1.1/1,000,000 py respectively, giving a male/female rate ratio of 4.5 (95%CI = 2.9–6.8). The rates of intentional and unintentional FIUI were 2 and 1/1,000,000 py respectively.

#### Intentional FIUI

The rates of intentional FIUI among males and females were 3.0 and 0.8/1,000,000 py respectively, giving a male/female rate ratio of 3.7 (95%CI = 2.1–6.3). Age-specific FIUI rates were 3.0, 3.0, 2.3, and 0.9 per 1,000,000 py for those aged 16–24, 25–34, 35–44, 45–64 respectively.

Controlling for age, females have a significantly lower risk of intentional FIUI than males (Rate ratio = 0.3, 95% CI = 0.2–0.5). Controlling for gender, the risk of intentional FIUI was similar in those aged 16–24, 25–34 and 35–44 years, but significantly lower in those aged 45 years and over compared to the referent group (those aged 16–24 years) (Table 3).

#### Unintentional FIUI

The rates of unintentional FIUI among males and females were 1.8 and 0.4/1,000,000 py respectively, giving a male/female rate ratio of 4.1 (95%CI = 2.0–8.5). Age-specific FIUI rates were 3.0, 1.8, 1.2, and 0.2 per 1,000,000 py for those aged 16–24, 25–34, 35–44, 45–64 years, respectively.

Controlling for age, females have a significantly lower risk of unintentional FIUI than males (Rate ratio = 0.3, 95% CI = 0.1–0.6). Controlling for gender, there was no significant difference in the risk of unintentional FIUI between those aged 16–24, 25–34 and 35–44 years. However, the risk of unintentional FIUI was significantly lower in those aged 45 years and over compared to the referent group (those aged 16–24 years) (Table 3).

### Ethnicity and FIUI

The majority of cases (79%) were White. Other ethnic groups were Black (8%), Asian 0.8% and Other 4.5%. Ethnicity was unknown in 8% of cases. The proportion of Black and Minority Ethnic (BME) groups among all FIUI cases (13%) in this study was higher than that in the standard population distribution of England (9.1%). However, the ethnicity distribution of intentional and unintentional FIUI was different. The risk of intentional FIUI among BMEs was higher than expected (Observed number = 14; Risk = 1.9, 95% CI = 1.1–3.1). The distribution in unintentional FIUI was unremarkable (Observed number = 14; Risk ratio = 0.7, 95% CI = 0.1–1.9.

## Discussion

This study has quantified the extent and pattern of fatal injuries while under the influence of psychoactive drugs. These fatalities account for 4% of all drug-related deaths in England, with a rate of 3/1000,000 person-years. However, this rate is likely to be an underestimate given that only sudden fatal incidents were reported to coroners. It could be argued that, as in other injury categories, many cases of FIUI may occur following a period of hospitalisation and consequently are not reported to the coroner. Rather, such cases are treated as deaths whose immediate causes are natural, with injury and drug abuse possibly reported as indirect or contributory causes.

Intentional and unintentional FIUI accounted for 96% of all cases. The leading mechanism of intentional FIUI was suffocation, present in 73% of these cases. This finding is consistent with the trend reported in England and Wales where suffocation is the most common method of suicide in men and the second most common in women [14]. In the United States, increase in the rate of suicide by suffocation among young people aged 15–19 years has been reported [15]. In Australia, suffocation, mainly by hanging, has remained the most common method of suicide in males since 1992, and the second most common method in females [16]. Altogether, our findings suggest that psychoactive substances play a significant role in intentional fatal injuries, especially hanging, possibly providing the 'courage' required for completing the act.

Road traffic accidents (RTAs) were the leading mechanism of unintentional FIUI. This finding is consistent with other studies of unintentional injury in general. In the US, Cohen, Miller, Sheppard, Gordon, Gantz and Atnafou [7] have showed that RTA accounted for the largest proportion (43%) of unintentional fatal injuries. Furthermore, alcohol-related RTA is known to account for 21% of fatal injuries in the European Union [17].

In both intentional (35%) and unintentional (26%) FIUI, alcohol-in-combination was the most frequently implicated psychoactive drug. The role of alcohol in injuries is well documented. However, what our study has revealed is that there is a need to examine the mechanism of the heightened vulnerability to fatal injuries when alcohol is consumed with other psychoactive drugs.

Furthermore, this study also shows gender and age variations in drug-specific FIUI. Alcohol-in-combination was the most frequently mentioned drug across all age groups. This is in contrast to reports on drug-related deaths in the UK where heroin consistently accounts for about 40% of all drug-related deaths [10,11]. However, there were variations in the pattern of involvement of other drugs across age groups. Next to alcohol-in-combination, cannabis was the most frequently mentioned drug in cases aged 16–24 years; cocaine in those aged 25–34 years; and sedative-hypnotics and antidepressants in the older age groups. This age variation in drug-specific fatal injuries is of immense value in developing targeted intervention strategies.

The implication of cannabis in 23% of unintentional FIUI cases challenges the general assumption that cannabis does not contribute to premature mortality like other illicit drugs. The proportion we reported is somewhat higher than the 12% reported by Turnbridge, Keigan and James [5] among cases of RTA fatalities alone. However, the mechanism of cannabis action in these fatalities would require further investigation given that cannabinoid metabolites are usually detectable in the tissue up to three weeks after consumption.

Another main finding is the identification of suffocation as the lead mechanism of FIUI, accounting for 47% of all cases analysed and about 90% of self-inflicted FIUI.

Comparisons between patterns fatal intentional and unintentional injuries while under the influence of psychoactive drugs are rarely examined in the injury literature. To that extent, the findings of this study, have provided the basis for further enquiry around the pharmacokinetics of psychoactive drugs and alcohol interaction and how these impact on fatal injury risks. This observation is pertinent because 42% of FIUI in this study involved drug interactions (34% in intentional and 61% in unintentional FIUI cases). When alcohol-in-combination is also considered, the percentage of cases of psychoactive drug interactions increase to 78%.

The role of ethnicity in fatal injuries has not been previously reported in the UK and is rarely reported elsewhere. One of the significant findings of this study is that in England, there is a heightened risk of intentional FIUI among Black and Minority Ethnic (BME) groups However, there is a need for further investigation of fatal injury risk determinants and modifiers in these groups.

One of the limitations of this study is the possible under-reporting of FIUI. There are two likely reasons for this. Firstly, the delay in the completion of coronial inquisition would mean that the actual frequency of FIUI would always be under-reported, given that it often takes months and sometimes years for a coroner to complete an inquest and reach a verdict. Therefore, data reporting often occurs in arrears. This study shares this limitation with other mortality studies that rely on coronial data.

Secondly, many FIUI cases may pass unreported because they are treated as deaths from natural causes if the cases died in a hospital or elsewhere after a period of treatment for 'non-fatal' injuries. In this respect, FIUI surveillance should include cases of sudden and delayed fatalities. However, the optimum duration of delay would need to be cautiously determined to ensure validity.

Thirdly, this study can at best be considered an exploratory investigation into FIUI in England, as it did not cover all coroner jurisdictions in England. However, the fact that the study covered 73% of coroner jurisdictions in England should attenuate the effects of any sampling bias.

Furthermore, the study did not report on the toxicological levels of the different psychoactive drugs detected at post-mortem. Over-reliance on the pathologist's report as a source of information is a limitation that this study shares with many studies of drug-related mortality. However, to have any meaning, report of toxicological levels should be accompanied by additional information on the individual case's potential for tolerance, history of drug abuse/dependence, psychiatric history and prescription pattern. Such studies would involve psychological autopsy, which is beyond the scope of this current investigation.

While the influence of drugs and alcohol on injury occurrence is well documented [3,7,8,18,19], little is known about the facilitative role of psychoactive drugs in fatal intentional and unintentional injuries, especially the role of drug-drug and alcohol-drug interactions. Therefore, the findings of this study have provided another path for developing evidence-based targeted prevention strategies.

## Conclusion

There are differences in the nature, extent and pattern of intentional and unintentional FIUI that should necessitate targeted prevention strategies. However, given that drug combinations with or without alcohol is the predominant pattern of consumption in intentional and unintentional FIUI, there is an opportunity for cross-discipline collaboration between injury prevention specialists and substance abuse/mental health specialists.

## Abbreviations

npSAD National Programme on Substance Abuse Deaths

FIUI Fatal Injury Under the Influence of Psychoactive Drugs

## Competing interests

The author(s) declare that they have no competing interests.

## Authors' contributions

AO wrote the manuscript, analysed the data and co-ordinated the study.

KC, RD and JC collected data and interpreted the results.

FS and HG were involved in study design, data interpretation and revising the article critically for intellectual content.

AO is the study guarantor.

All authors read and approved the final manuscript.

## Pre-publication history

The pre-publication history for this paper can be accessed here:



## Figures and Tables

**Figure 5:**
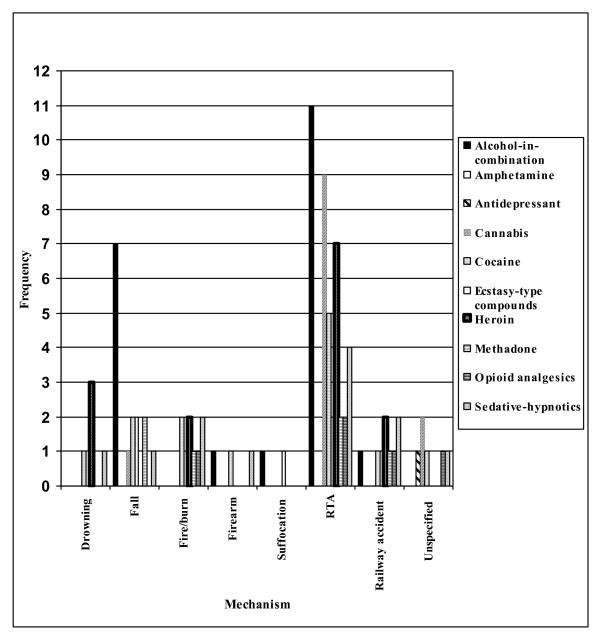
Unintentional FIUI mechanisms and implicated drugs.

**Table 1:** Pattern of drug combinations in intentional and unintentional FIUI

**Drug combinations**	**Intentional FIUI**	**Unintentional FIUI**
Amphetamine+alcohol	1	0
Antidepressant+alcohol	1	0
Antidepressant+hypnotics	2	0
Antidepressants+opioids+hypnotics+cannabis	0	1
Cannabis +alcohol	7	4
Cannabis+amphetamines+alcohol	1	0
Hypnotics+alcohol	2	0
Opioids+alcohol	1	0
Ecstasy + alcohol	1	3
Ecstasy+amphetamine	1	0
Cocaine +cannabis	2	1
Cocaine+alcohol	1	4
Cocaine+ecstasy	1	0
Cocaine+ecstasy+amphetamine+alcohol	1	0
Cocaine+cannabis+alcohol	0	1
Cocaine+hypnotics+alcohol	0	1
Opioids+hypnotics+cannabis	0	1
Methadone+cannabis+alcohol	0	1
Methadone+hypnotics+alcohol	0	1
Methadone+opioids+hypnotics+alcohol	1	0
Methadone+cocaine+hypnotics	1	0
Heroin+hypnotics	0	3
Heroin+alcohol	1	0
Heroin+cocaine	0	1
Heroin+hypnotics+cannabis+alcohol	1	0
Heroin+opioids+cannabis+alcohol	0	1
Heroin+opioids+hypnotics	1	0
Heroin+opioids+hypnotics+alcohol	0	1
Heroin+cocaine+amphetamine	1	0
Heroin+cocaine+ecstasy	0	1
Heroin+cocaine+hypnotics	0	1
Heroin+cocaine+opioids+hypnotics	0	2
Heroin+antidepressants	1	0
Heroin+methadone	0	1
Heroin+methadone+alcohol	0	1
Heroin+methadone+hypnotics	1	0
Heroin+methadone+opiods+alcohol	1	0
Heroin+methadone+cocaine+hypnotics	0	1
	31	31

**Table 2:** Differences in drugs mentioned in fatality between FIUI and non-FIUI cases

	**Intentional**			**Unintentional**		
**Drugs implicated**	**% FIUI cases (N = 90)**	**% Non-FIUI cases (Intentional poisoning only) cases (N = 604)**	**MOR (95%CI)**	**% FIUI cases (N = 52)**	**% Non-FIUI cases (Unintentional poisoning) (N = 2681)**	**MOR (95%CI)**

Alcohol-in-combination	35.2	22.8	1.5 (1.1–2.1)	41.2	25.8	1.6 (1.1–2.2)
Amphetamines	5.7	1.2	4.9 (1.6–15.2)	0	2.3	-
Antidepressants	12.5	35.0	0.4 (0.2–0.6)	10.3	2.0	0.2 (0.03–1.3)
Cannabis	15.9	0	-	23.5	1.3	18.6 (10.2–38.7)
Cocaine	9.1	0.8	11.0 (3.7–32.9)	25.5	5.9	4.3 (2.6–7.0)
Ecstasy-type drugs	4.5	0.8	5.5 (1.5–20.1)	9.8	2.3	4.3 (1.8–10.3)
Heroin	9.1	5.6	1.6 (0.8–3.4)	31.4	90.1	0.6 (0.4–0.9)
Sedative-hypnotics	14.8	15.5	1.0 (0.6–1.6)	23.5	11.7	2.0 (1.2–3.3)
Methadone	4.5	3.0	1.5 (0.5–4.4)	11.9	14.1	0.8 (0.4–1.8)
Opioid analgesics	5.7	36.3	0.2 (0.06–0.4)	9.8	11.5	0.9 (0.4–2.0)

**Table 3:** Age and gender effects on FIUI

	**Intentional FIUI**			**Unintentional FIUI**		
**Variable**	**Number of FIUI**	**Rate ratio**	**95% CI**	**Number of FIUI**	**Rate ratio**	**95% CI**

**Main effects**						
Gender						
Male	73	1	-	43	1	-
Female	17	0.27	0.16–0.46	9	0.24	0.12–0.50

Age						
16–24	14	1		14	1	-
25–34	33	0.99	0.53–1.86	20	0.60	0.31–1.20
35–44	26	0.75	0.39–1.44	14	0.41	0.19–0.85
45–64	17	0.31	0.15–0.63	4	0.07	0.02–0.22

**Controlling for age**						
Gender						
Male	73	1		43	1	-
Female	17	0.27	0.16–0.47	9	0.25	0.07–0.87

**Controlling for gender**						
Age						
16–24	14	1	-	14	1	-
25–34	33	1.48	0.79–2.79	20	0.89	0.44–1.79
35–44	26	1.11	0.58–2.15	14	0.60	0.28–1.27

45–64	17	0.46	0.22–0.93	4	0.11	0.04–0.33
